# Clinical and histopathological characteristics and survival analysis of 4594 Japanese patients with melanoma

**DOI:** 10.1002/cam4.2110

**Published:** 2019-04-01

**Authors:** Yasuhiro Fujisawa, Shusuke Yoshikawa, Akane Minagawa, Tatsuya Takenouchi, Kenji Yokota, Hiroshi Uchi, Naoki Noma, Yasuhiro Nakamura, Jun Asai, Junji Kato, Susumu Fujiwara, Satoshi Fukushima, Jiro Uehara, Toshihiko Hoashi, Tatsuya Kaji, Taku Fujimura, Kenjiro Namikawa, Manabu Yoshioka, Naoki Murao, Dai Ogata, Kanako Matsuyama, Naohito Hatta, Yoshitsugu Shibayama, Toshiharu Fujiyama, Masashi Ishikawa, Daisuke Yamada, Akiko Kishi, Yoshiyuki Nakamura, Takatoshi Shimiauchi, Kazuyasu Fujii, Manabu Fujimoto, Hironobu Ihn, Norito Katoh

**Affiliations:** ^1^ Japanese Melanoma Study Group Tsukuba Japan; ^2^ Prognosis and Statistical Investigation Committee of the Japanese Skin Cancer Society Kumamoto Japan; ^3^ Department of Dermatology University of Tsukuba Tsukuba Japan; ^4^ Department of Dermatology Shizuoka Cancer Center Shizuoka Japan; ^5^ Department of Dermatology Shinshu University School of Medicine Matsumoto Japan; ^6^ Department of Dermatology Niigata Cancer Center Niigata Japan; ^7^ Department of Dermatology University of Nagoya Nagoya Japan; ^8^ Department of Dermatology University of Kyushu Fukuoka Japan; ^9^ Department of Dermatology Osaka City University Osaka Japan; ^10^ Department of Dermatology Saitama Medical University International Medical Center Hidaka Japan; ^11^ Department of Dermatology Kyoto Prefectural University of Medicine Kyoto Japan; ^12^ Department of Dermatology Sapporo Medical University Sapporo Japan; ^13^ Department of Dermatology Kobe University Kobe Japan; ^14^ Department of Dermatology and Plastic Surgery Kumamoto University Kumamoto Japan; ^15^ Department of Dermatology Asahikawa Medical University Asahikawa Japan; ^16^ Department of Dermatology, Nippon Medical School Bunkyo‐ku Japan; ^17^ Department of Dermatology Okayama University Graduate School of Medicine Okayama Japan; ^18^ Department of Dermatology Tohoku University Graduate School of Medicine Sendai Japan; ^19^ Department of Dermatologic Oncology National Cancer Center Hospital Tokyo Japan; ^20^ Department of Dermatology University of Occupational and Environment Health Kitakyushu Japan; ^21^ Department of Plastic Surgery University of Hokkaido Sapporo Japan; ^22^ Department of Dermatology Saitama Medical University Hidaka Japan; ^23^ Department of Dermatology University of Gifu Gifu Japan; ^24^ Department of Dermatology Toyama Prefectural Central Hospital Toyama Japan; ^25^ Department of Dermatology Fukuoka University Fukuoka Japan; ^26^ Department of Dermatology Hamamatsu University School of Medicine Hamamatsu Japan; ^27^ Department of Dermatology Saitama Prefectural Cancer Center Saitama Japan; ^28^ Department of Dermatology University of Tokyo Tokyo Japan; ^29^ Department of Dermatology Toranomon Hospital Minato‐ku Japan; ^30^ Department of Dermatology Kagoshima University Kagoshima Japan

**Keywords:** acral, Asian, Epidemiology, Japanese, Melanoma, mucosal

## Abstract

**Background:**

The incidence of melanoma among those of an Asian ethnicity is lower than in Caucasians; few large‐scale Asian studies that include follow‐up data have been reported.

**Objectives:**

To investigate the clinical characteristics of Japanese patients with melanoma and to evaluate the prognostic factors.

**Methods:**

Detailed patient information was collected from the database of Japanese Melanoma Study Group of the Japanese Skin Cancer Society. The American Joint Committee on Cancer seventh Edition system was used for TNM classification. The Kaplan‐Meier method and Cox proportional hazards model were used to estimate the impact of clinical and histological parameters on disease‐specific survival in patients with invasive melanoma.

**Results:**

In total, 4594 patients were included in this analysis. The most common clinical type was acral lentiginous melanoma (40.4%) followed by superficial spreading melanoma (20.5%), nodular melanoma (10.0%), mucosal melanoma (9.5%), and lentigo maligna melanoma (8.1%). The 5‐year disease‐specific survival for each stage was as follows: IA = 98.0%, IB = 93.9%, IIA = 94.8%, IIB = 82.4%, IIC = 71.8%, IIIA = 75.0%, IIIB = 61.3%, IIIC = 41.7%, and IV = 17.7%. Although multivariate analysis showed that clinical classifications were not associated with survival across all stages, acral type was an independent poor prognostic factor in stage IIIA.

**Conclusions:**

Our study revealed the characteristics of melanoma in the Japanese population. The 5‐year disease‐specific survival of each stage showed a similar trend to that of Caucasians. While clinical classification was not associated with survival in any stages, acral type was associated with poor survival in stage IIIA. Our result might indicate the aggressiveness of acral type in certain populations.

## 1 INTRODUCTION

In Japan, melanoma is classified as “rare cancer,” but the precise melanoma incidence in Japan has not been well investigated. Analysis of the Hospital Based Cancer Registries in Japan revealed the crude annual incidence of melanoma to be 1.75 per 100 000 people^1^; whereas the data from the World Health Organization showed the incidence of melanoma in Japan to be between 0.3 and 0.8 per 100 000 people (male: 0.3‐0.8; female: 0.4‐0.6).^2^ According to the Surveillance, Epidemiology, and End Results Program (SEER) data, Asian and Pacific Islanders had an annual incidence of 1.68 melanoma patients per 100 000 people in contrast to that of non‐Hispanic Whites at 33.85 per 100 000 people,^3^ clearly showing the lower incidence among Asians. Due to rarity of melanoma in Japan, it has been difficult to accumulate a large enough volume of patient data to conduct statistical analysis for investigating the unique characteristics of melanoma occurrence in the Japanese population.

In 2001, Ishihara and colleagues reported the results of a nationwide survey to investigate melanoma incidence among Japanese patients.^4^ In the 2008 update of their study,^5^ a survival analysis of 2065 Japanese patients with melanoma, Ishihara and colleagues reported that the survival of acral lentiginous melanoma (ALM), which constituted 47% of their cohort, was statistically worse than that of superficial spreading melanoma (SSM). However, their analysis was based on the Kaplan‐Meier method and log‐rank test and did not include multivariate analysis to compensate for other factors such as TNM status. Recently, molecular characterizations of ALM and mucosal melanoma (MCM) have shown that these clinical types are biologically distinct from the more common cutaneous melanomas.^6^, ^7^ Some reports indicated that these two subtypes correlated with poor survival,^8^, ^9^, ^10^, ^11^ while others have reported no correlation between clinical subtypes and prognosis.^12^, ^13^

The recent development of immune checkpoint inhibitors has dramatically changed the treatment of melanoma.^14^, ^15^, ^16^ The response to immune checkpoint inhibitor among Japanese patients, however, has not been as favorable as that of Caucasians. For example, in Japanese clinical trials of anti‐programmed death receptor‐1 (PD‐1) monoclonal antibody, the response ratios were reported to be 24.1%^17^ to 34.8%^18^ compared to 38%^16^ to 43.7%^19^ from studies conducted in the West, indicating that the Japanese patients with melanoma might have distinct PD‐L1 expression profiles.

In the current study, we collated and analyzed nationwide patient data (including data on survival) to investigate the details of melanoma among the Japanese population and performed multivariate analyses to reveal the factors associated with survival by clinical type.

## 2 MATERIALS AND METHODS

The Committee of Statistical Survey of Skin Cancer Prognosis of the Japanese Skin Cancer Society are currently conducting a survey named the “Japanese Melanoma Study (JMS)” to accumulate data of patients with melanoma admitted to 27 institutes in Japan since 2005 (See Table S1 for the collaborating institutes). The JMS currently has an online patient information submission system and collaborating researchers access the web system at least once a year to submit new patient information and also update prognostic information and additional treatment performed after any recurrence. The information of patient collected from 2005 to 2017 is described in Table S1. In brief, age, sex, site of the primary tumor, clinical type, Breslow thickness, Clark level, regression, in‐transit/satellite, the American Joint Committee on Cancer (AJCC) seventh Edition TNM status, and follow‐up data including type of recurrence and prognosis were included in the survey. All data pertaining to vital status and date of death were solely dependent on the investigator's reports. Patient follow‐up was conducted according to each doctor's decision, generally following guidelines published by the Japanese Skin Cancer Society or National Comprehensive Cancer Network (NCCN). In the Japanese guideline, the recommendation is as follows: at least every 3 months for the first year and every 6 months for years 2‐5 for stage I; every 3 months for the first 3 years and every 6 months for years 4‐5 for stage II; and every month for the first 3 years and every 3 months for years 4‐5 for stage III or more. The follow‐up interval in NCCN is longer than the Japanese guideline. The follow‐up interval might be longer than the recommendation for elderly patients. This study was approved by the University of Tsukuba Hospital (59‐1) and the Japanese Dermatology Association.

Chi‐squared test was used to calculate *P*‐values of categorical difference. Kaplan‐Meier method was used to estimate survival curves and log‐rank test was used to calculate the corresponding *P*‐values. Cox proportional hazard model was used for both univariate and multivariate analysis to calculate factors associated with survival. First, we tested using a univariate analysis for factors that may associate with outcome. Next, in the multivariate analysis, we included factors that were shown to be statistically significant in the univariate analysis. All statistical analysis was performed using free statistical analysis software R and *P *< 0.05 were considered as significant.

## 3 RESULTS

### 3.1 Primary tumor

In total, data for 4594 patients were submitted to this survey. Background data are given in Table 1. The participants were 54.1% women and the average age of entire cohort was 64.1 years. As shown in Table 2, the most common site of the primary tumor was the lower extremity (41.7%), followed by the upper extremity (20.2%), head and neck (14.2%), trunk (14.8%), and mucosa (9.5%). Of note, 44.7% of all melanoma occurred in hands and feet. Accordingly, the most common clinical type was ALM (40.4%), followed by SSM (20.5%), nodular melanoma (NM, 10.0%), mucosal melanoma (MCM, 9.5%), and lentigo maligna melanoma (LMM, 8.1%).

**Table 1:** Background of the study cohort

	Number	% of Total
Total number	4594	
Sex		
Male	2107	45.9%
Female	2484	54.1%
Not described	3	
Age (years)		
Average	64.1	
Median	67.0	
Range	2‐103	
Pregnancy when diagnosed		
Yes	16	0.6%
No	1557	
Not described	911	
Familial melanoma history		
Yes	97	2.1%
No	2576	
Not described	1921	
History of other malignancy		
Yes	361	7.9%
No	2475	
Not described	1758	

**Table 2:** Site of the primary tumor and clinical type of the entire study cohort (n = 4594)

	Number	Percent	
Site of the primary			
Head and neck	652	14.2%	
Trunk	682	14.8%	
Upper extremity			
Hand	533	11.6%	20.2%
Other than hand	211	8.6%	
Lower extremity			
Foot	1522	33.1%	41.7%
Other than foot	394	8.6%	
Mucosa	438	9.5%	
Uveal	45	1.0%	
Primary unknown	99	2.2%	
Not described	41	0.9%	
Clinical type			
Acral lentiginous	1857	40.4%	
Superficial spreading	942	20.5%	
Nodular	458	10.0%	
Lentigo maligna	372	8.1%	
Mucosal melanoma	438	9.5%	
Primary unknown	99	2.2%	
Others/not described	383	9.3%	

Ulceration was present in 29.1% (n = 3629) of ALM, SSM, NM, and LMM tumors (four clinical types, Table 3) and regression in 3.4%. Average Breslow thickness was 3.73 mm with median of 2.20 mm. T classification based on AJCC seventh Edition in patients with complete pathological staging (n = 3188) is as follows (Table 4): Tis = 23.6%, T1a = 16.2%, T1b = 2.4%, T2a = 10.1%, T2b = 3.3%, T3a = 8.7%, T3b = 9.3%, T4a = 7.1%, T4b = 18.1%, and not assessed = 1.3%. For reference, data of TNM status of AJCC database^20^ are included in the Table 4. The distribution of the T subclass was significantly different compared with the AJCC database; the proportion of higher T status was more frequent in our study population.

**Table 3:** Characteristics of primary tumor (n = 3629, only with four clinical classifications)

	Number	% of Total
Ulceration		
Yes	1055	29.1%
No	2429	66.9%
Not described	145	4.0%
Regression		
Yes	125	3.4%
No	2026	55.8%
Not described	1218	40.8%
Clark's classification		
1	649	17.9%
2	338	9.3%
3	409	11.3%
4	794	21.9%
5	414	11.4%
Not described	1023	28.2%
Breslow's thickness		
Average	3.73 mm	
Median	2.20 mm	

**Table 4:** TNM classification (AJCC 2009, only with four clinical classifications)

	Current study			AJCC data	*P*‐value
	Number	% of Total	% Among patients with T1a‐T4b		
T status					
Tis	753	23.6%			
T1a	518	16.2%	21.6%	34.2%	*P* < 0.000001 chi‐square test
T1b	75	2.4%	3.1%	8.6%	
T2a	321	10.1%	13.4%	5.5%	
T2b	105	3.3%	4.4%	23.6%	
T3a	277	8.7%	11.6%	11.3%	
T3b	298	9.3%	12.4%	7.8%	
T4a	225	7.1%	9.4%	3.8%	
T4b	576	18.1%	24.1%	5.1%	
Not available	40	1.3%			

	Number	% of Total	% Among patients with N1a‐N3		
N status					
0	2249	70.5%			
N1a	259	8.1%	29.1%	44.3%	*P* < 0.000001 chi‐square test
N1b	81	2.5%	9.1%	4.7%	
N2a	154	4.8%	17.3%	17.4%	
N2b	82	2.6%	9.2%	4.6%	
N2c	38	1.2%	4.3%	12.1%	
N3	275	8.6%	30.9%	16.9%	
Not available	50	1.6%			

	Number	% of Total			
M status					
0	2990	93.8%	6.2%		
M1	46	1.4%			
M1a	43	1.3%			
M1b	79	2.5%			
M1c	30	0.9%			

### 3.2 Lymph node status

Sentinel lymph node (SLN) biopsy was performed in 2076 (65.1%) of patients with ALM, SSM, NM, or LMM and 481 (23.1%) of them involved metastasis. Lymph node dissection without SLN biopsy was performed in 288 (9.0%) patients and 203 (70.5%) had tumor in the dissected nodes. Satellite or in‐transit metastasis was present in 151 (4.7%) patients. N classification is as follows (Table 4): N0 = 70.5%, N1a = 8.1%, N1b = 2.5%, N2a = 4.8%, N2b = 2.6%, N2c = 1.2%, N3 = 8.6%, and not assessed = 1.6%. For reference, TNM status of AJCC database^20^ was included in Table 4. The number of patients with N3 disease in our study population was significantly higher compared with the AJCC database.

### 3.3 Distant metastasis

As described in Table 4, 198 (6.2%) patients with ALM, SSM, NM, and LMM had metastasis at diagnosis. Of them, 79 (40.0%) of those had elevated serum LDH levels (M1c).

### 3.4 Pathological TNM staging and 5‐year disease‐specific survival

**Figure 1:**
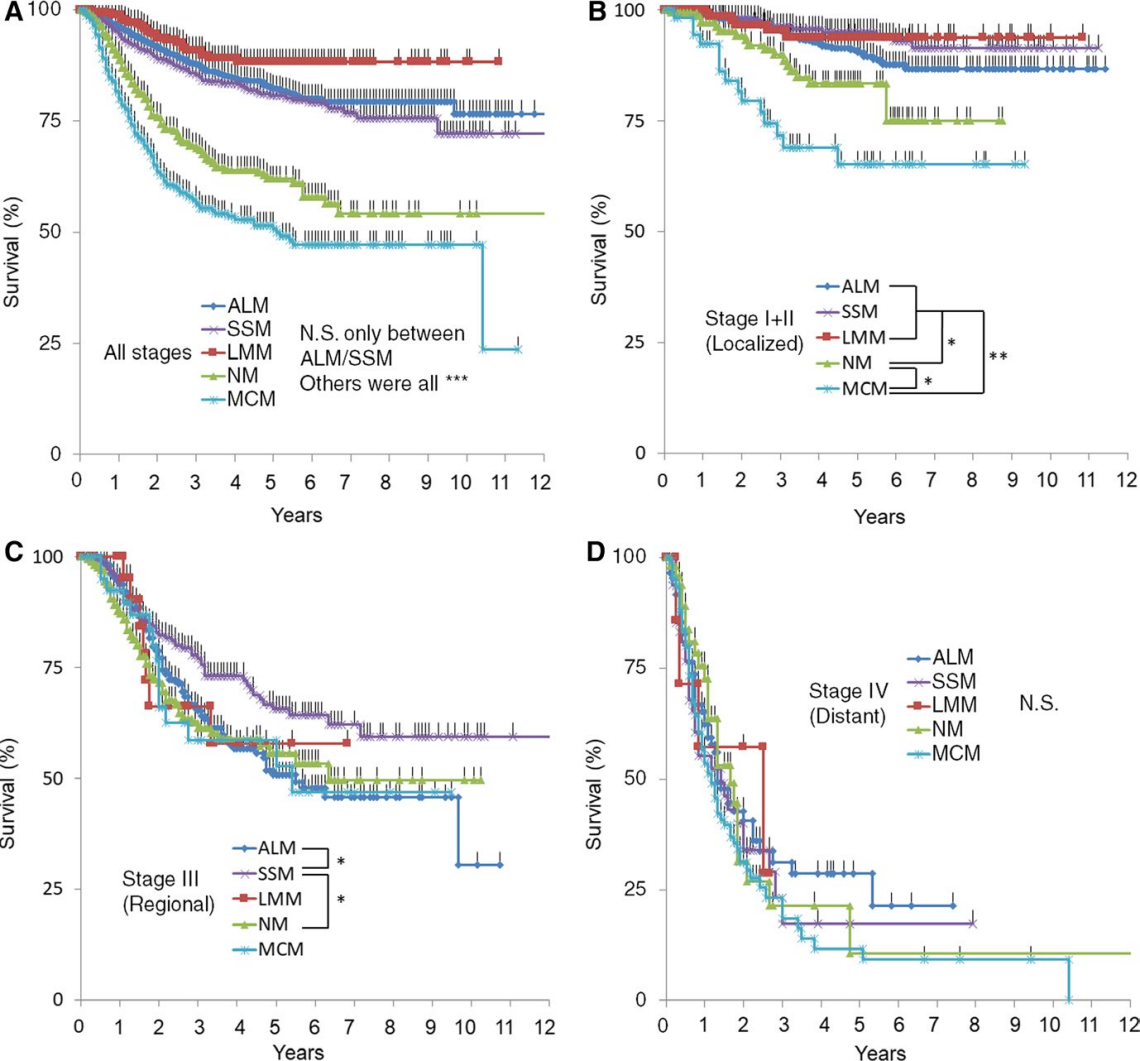
Kaplan‐Meier plots for patients classified according to the AJCC version seventh TNM classification system. Only patients with four clinical types (ALM, SSM, LMM, and NM) and have pathological diagnosis were included in this analysis. Survival curves for (A) all stages; (B) Stages I and II; (C) Stage III; and (D) Stage IV. * *P* < 0.05, ** *P* < 0.01, *** *P* < 0.001

Data of pathological primary tumor and LN evaluation were available for 3438 patients, which included ALM, SSM, NM, LM, and MCM. Kaplan‐Meier (K‐M) plots for these five tumor types are shown in Figure 1A. Of these 3438 patients, follow‐up was lost for 465 of them during the study period. NM and MCM had significantly worse survival compared with other tumor types. There was no survival difference in between ALM and SSM. Next, after excluding patients with tumor in situ (Tis), we classified the remaining patients by localized, regional, and distant disease. K‐M plots for each category are shown in Figure 1B‐D. MCM showed a significantly lower survival in localized disease, but did not reach statistical significance in regional disease. Survival of patients with NM was statistically worse in both localized and regional disease. Interestingly, patients with ALM with regional disease showed poor survival compared with SSM.

**Figure 2:**
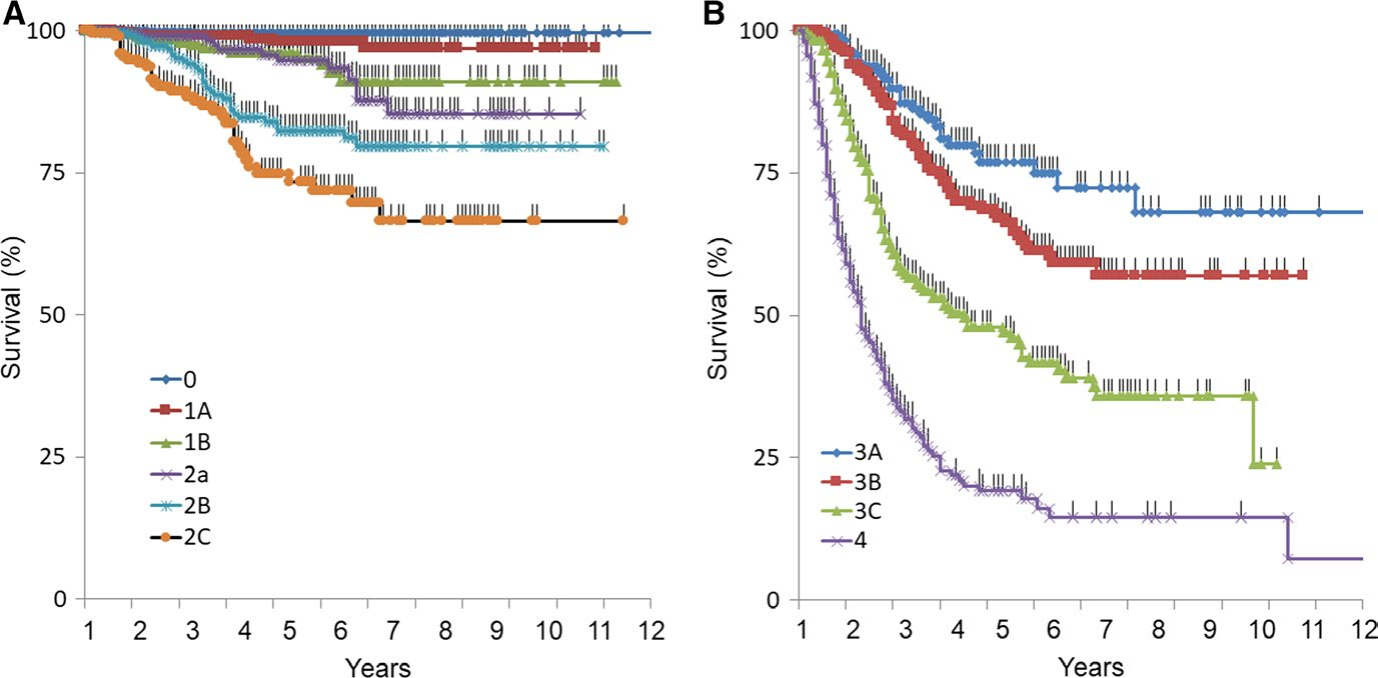
Kaplan‐Meier plots for each stages. (A) Survival curves for stage 0, 1 and 2. (B) Survival curves for stage 3 and 4

Next the 3199 patients with ALM, SSM, NM, and LMM were classified according to the AJCC seventh Edition staging system and the result were as follows: stage 0 = 23.3%, IA = 15.2%, IB = 9.9%, IIA = 7.5%, IIB = 8.2%, IIC = 5.7%, IIIA = 5.7%, IIIB = 9.7%, IIIC = 8.7%, and IV = 6.2% (Table 5). Therefore, localized disease constituted 69.7%, regional disease 24.0%, and distant disease 6.2% of the total melanoma. The 5‐year disease‐specific survival (DSS) rate was as follows: IA = 98.0%, IB = 93.9%, IIA = 94.8%, IIB = 82.4%, IIC = 71.8%, IIIA = 75.0%, IIIB = 61.3%, IIIC = 41.7%, and IV = 17.7%. For reference, TNM status of AJCC database^20^ is included in Table 5. The distribution of TNM stage was significantly different compared with AJCC. K‐M plots for each substage are shown in Figure 2A,B. Separation of survival curves within each stage was clear, indicating that the AJCC TNM staging system could clearly classify the melanoma of Japanese patients.

**Table 5:** Pathological TNM classification (AJCC seventh edition, only with four clinical classifications)

Stage	Number	% of Total	% Among patients without Tis				5‐Year DSS rate (%)		
	Current study				AJCC data	*P*‐value	Current study		AJCC data
0	743	23.3%					99.6%		
IA	483	15.2%	19.8%		24.5%	*P* < 0.000 001 chi‐square test	98.0%		97%
IB	316	9.9%	12.9%		23.1%		93.9%		94%(T1b)/91%(T3a)
IIA	238	7.5%	9.7%		12.0%		94.8%		82%(T2b)/79%(T3a)
IIB	260	8.2%	10.6%		8.4%		82.4%		68%(T3b)/71%(T4a)
IIC	182	5.7%	7.4%		3.6%		71.8%		53%
IIIA	181	5.7%	7.4%		3.1%		75.0%		78%
IIIB	309	9.7%	12.6%		3.6%		61.3%		59%
IIIC	278	8.7%	11.4%		1.9%		41.7%		40%
IV	198	6.2%	8.1%		19.8%		17.7%		14%
Total	3188	100%							

### 3.5 Factors associated with survival

To investigate the factors associated with survival, only the patient data that included pathological classification and survival data were used. After excluding patients with Tis, 2990 patients with invasive melanoma data were used for the further analysis. The result summary is shown in Table 6. All the factors in this table have been reported to associate with survival. Besides all the factors included in the univariate analysis shown to be statistically significant prognostic factors, higher age (hazard ratio [HR]=1.01, *P* < 0.05), T status (HR = 1.552, *P* < 0.000001), N status (HR = 1.776, *P* < 0.000001), and distant metastasis (HR = 3.331, *P* < 0.000001) were shown to be independent prognostic factors for survival.

**Table 6:** Factors associated with survival in all stages determined by Cox regression model (n = 2990)

	Univariate analysis				Multivariate analysis			
	HR	95% CI low	95% CI high	*P*‐value	HR	95% CI low	95% CI high	*P*‐value
Age (cont)	1.004	0.998	1.009	0.203	**1.01**	**1.004**	**1.016**	**0.001 21**
Sex (male:0)	0.6194	0.5101	0.7521	0.000 001	0.8652	0.6652	1.125	0.280
T status (cont)	2.415	2.173	2.684	0.000 000	**1.552**	**1.335**	**1.803**	**<0.000 00**
Ulceration	3.438	2.811	4.206	<0.000 00	1.256	0.9344	1.689	0.131
Regression	1.567	1.031	2.38	0.0354	1.289	0.7783	2.134	0.324
Vitiligo	2.303	1.088	4.876	0.0293	1.954	0.7589	5.032	0.165
ALM (ref:SSM)	0.7244	0.597	0.8791	0.001 09	0.8605	0.6227	1.189	0.363
LMM (ref:SSM)	0.4259	0.2656	0.683	0.0004	0.5179	0.2216	1.211	0.129
NM (ref:SSM)	2.63	2.1	3.294	<0.000 00	1.379	0.9547	1.993	0.0867
Satellite/in‐transit	5.98	4.625	7.733	<0.000 00	1.025	0.6996	1.502	0.899
N status (cont)	2.602	2.402	2.818	<0.000 00	**1.776**	**1.553**	**2.03**	**<0.000 00**
Distant metastasis	11.83	9.444	14.82	<0.000 00	**3.331**	**2.314**	**4.796**	**<0.000 00**

ALM, acral lentiginous melanoma; CI, confidence interval; Cont, continuous variable; HR, hazard ratio; NC, not computable; N, nodal; ref, reference; SSM, superficial spreading melanoma, T, tumor thickness

Bold represents factors below *P* < 0.05 in multivariate analysis

ALM and MCM have been reported to be associated with poor survival. We chose to focus on ALM, because MCM had already been shown to be a poor prognostic factor in the analysis using entire cohort (Figure 1). As shown in Figure 1A, no statistical difference was found only between ALM and SSM. Investigation of the survival curves of localized and regional disease classified by clinical type (Figure 1B), revealed that MCM and NM had worse survival compared with other types in localized disease (*P* < 0.05 and <0.0001, respectively). In regional disease (stage III), ALM and NM showed worse survival compared with SSM (Figure 1C, both *P* < 0.05). There was no statistical difference between clinical types in stage IV (Figure 1D).

**Figure 3:**
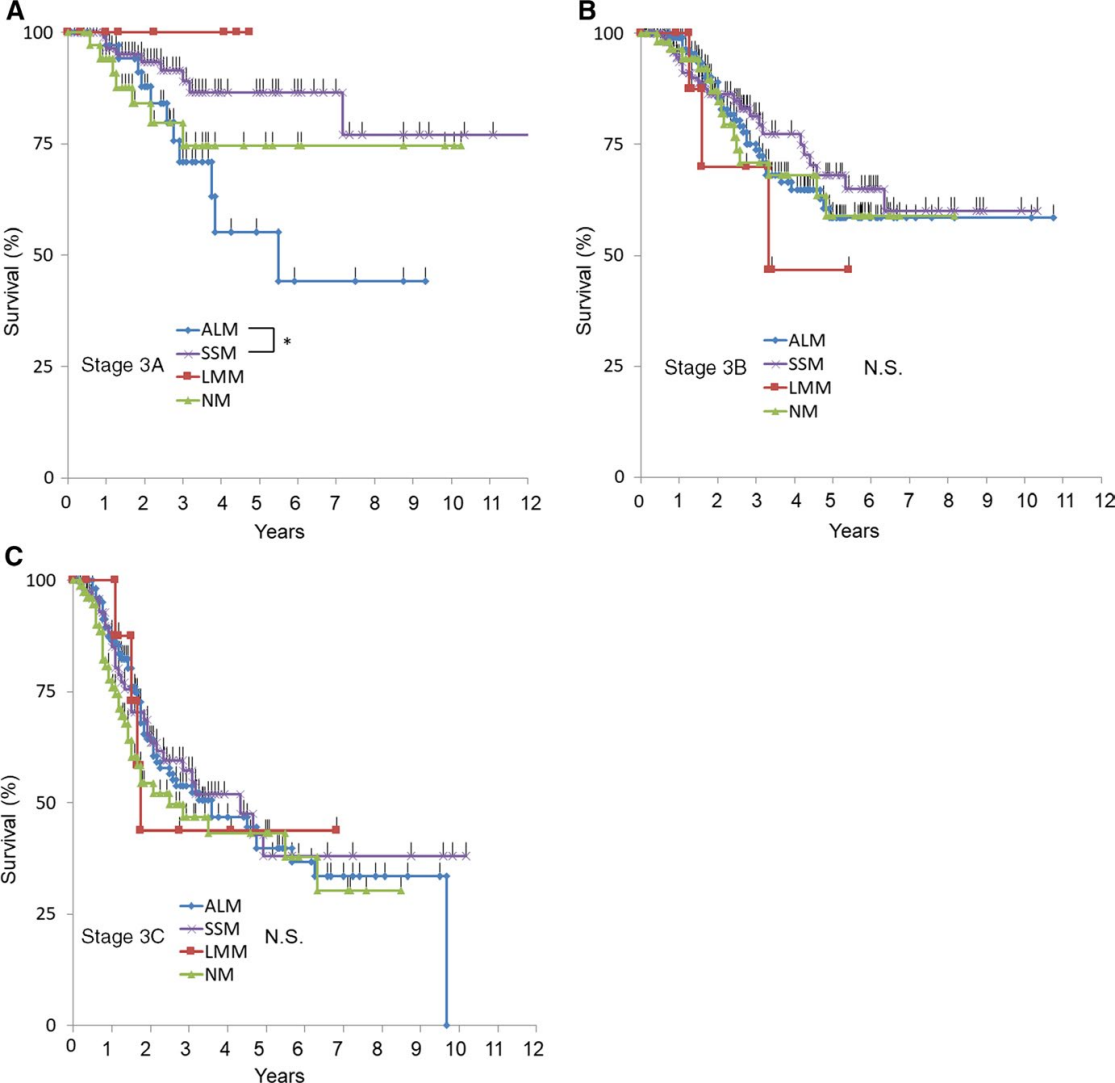
Kaplan‐Meier plots for Stage 3A‐3C. *P < 0.05

Next, each sub‐stage of stage III was further investigated. As shown in Figure 3A,C, stages IIIB and IIIC showed no survival difference between clinical types; however, survival of ALM was worse than that of SSM in stage IIIA. As T status and ALM were shown to be statistically significant (*P* < 0.05) in the univariate analysis, we included these factors in the multivariate analysis. As a result, multivariate analyses (Table 7) revealed that only ALM (HR = 2.80, *P* < 0.05) and T status (HR = 1.26, *P* < 0.01) were associated with poor survival in stage IIIA. We suspected the deviation of T and N status between ALM and SSM might explain the survival difference; however, the proportion of T1a‐4a and N1a‐2a in both groups was similar (data not shown). Therefore, no factor that could explain this survival difference apart from clinical type could be found. However, the number of patients in stage IIIA was small (ALM = 45, LMM = 7, NM = 36, and SSM = 92), and therefore, this difference might be just coincidental. Therefore, we need to accumulate more patient data to confirm the results.

**Table 7:** Factors associated with survival in stage IIIA determined by Cox regression model (n = 180)

	Univariate analysis				Multivariate analysis			
	HR	95% CI low	95% CI high	*P*‐value	HR	95% CI low	95% CI high	*P*‐value
Age (cont)	0.995	0.972	1.02	0.686				
Sex (male: 0)	1.10	0.513	2.36	0.808				
T status (cont)	2.02	1.26	3.24	0.003 41	**2.26**	**1.366**	**3.734**	**0.0015**
Ulceration	0.805	0.109	5.96	0.831				
ALM (ref: SSM)	2.36	1.092	5.09	0.029	**2.96**	**1.349**	**6.474**	**0.0068**
LMM (ref: SSM)	NC							
NM (ref: SSM)	1.37	0.58	3.25	0.471				
N2a (ref: N1a)	1.40	0.649	3.02	0.391				

ALM, acral lentiginous melanoma; CI, confidence interval; Cont, continuous variable; HR, hazard ratio; NA, not applicable; NC, not computable; ref, reference; SSM, superficial spreading melanoma

Bold represents factors below *P* < 0.05 in multivariate analysis

**Figure 4:**
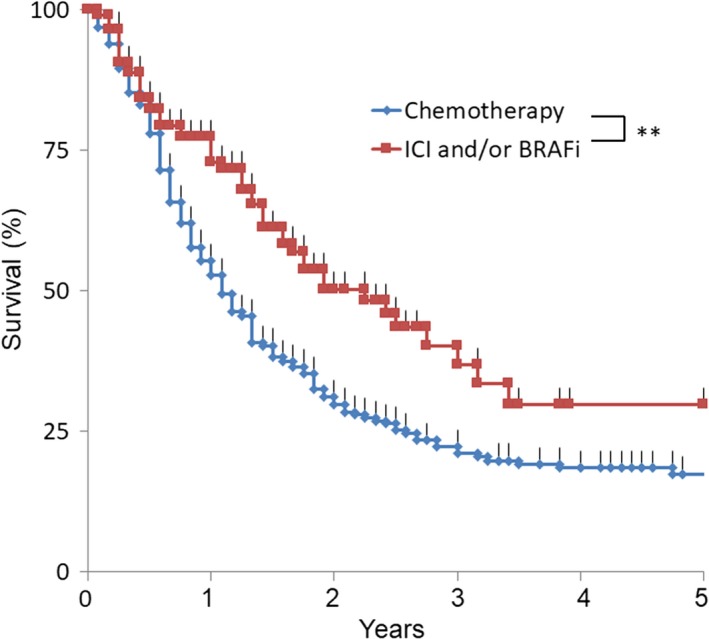
Kaplan‐Meier plots for Stage 4. Patients who received ICI and/or BRAFi had prolonged survival compared with those who did not. BRAFi, BRAF inhibitor; ICI, immune checkpoint inhibitors. ** *P* < 0.01

In Japan, nivolumab treatment first became available in 2014, thus, aside from those who patients involved in the clinical trials, we have only 4 years of experience with this treatment. As shown in Figure 4, there was a survival advantage in patients with stage IV who received immune checkpoint inhibitors and/or BRAF inhibitors (*P* < 0.001), clearly showing the benefit of these new drugs.

## 4 DISCUSSION

Our study revealed the unique characteristic of Japanese melanoma patients. Notably, the most common clinical type was ALM (40.4%); in contrast, the proportion of ALM among non‐Hispanic whites in the SEER database was only 1.5%.^3^ Therefore, 44.7% of all melanoma developed in the hand or foot. Furthermore, because 9.5% of those were MCM, ALM, and MCM together account for nearly half of all melanoma in Japan while SSM, the most common clinical type among Caucasian was only 20.5%. This fact clearly shows that the Japanese and Western melanoma patient populations differ greatly in terms of the most common clinical types. This fact makes a difference in the proportion of cases with BRAF mutation; ALM and MCM are known to harbor the BRAF mutation far less than SSM.^6^ Indeed, Uhara and colleagues reported that among 171 Japanese melanoma patients, overall BRAF mutation frequency was only 30.4% due to the low frequency of the mutation in ALM and MCM.^21^ In their study, only 12.5% of nail bed, 8.9% of palm or sole, and none of the MCM patients had the BRAF mutation.^21^ Thus, nearly 70% of advanced melanoma that occur in the Japanese population would have no benefit from BRAF inhibitor and this is a great disadvantage when compared with Western countries that have more (>50%)^22^ BRAF mutations.

Japanese melanoma patients tended to have considerably thicker primary tumors as shown in Table 4. The proportion of T4b in Japanese patients was 27.8%, whereas the AJCC database reported only 5.1%.^20^ Similarly, the proportion of patients with N3 was higher than in the AJCC database (Table 4, 30.9% and 16.9%, respectively). As a result, the Japanese population has a greater proportion of higher stage melanoma; 27.4% of patients were stage III, compared with only 8.6% reported in the AJCC database. These findings suggest that, because of its rarity, not many people in the general public are aware of melanoma and consequently they tended not to visit a dermatologist at early stages of the disease.

When comparing the 5‐year survival in our study with the AJCC database^20^ (Table 5), we found a difference of nearly 20% for stage IIC; 71.8% and 53%, respectively. Similarly, survival of stage IIA and IIB in our study was 93.9% and 82.4%, respectively, compared to 82% (T2b), 79% (T3a), 68% (T3b), and 71% (T4a) in the AJCC database. On the other hand, substages of stage III were similar; IIIA, IIIB, and IIIC in our study were 78.8%, 62.2%, and 42.3%, respectively, compared to 78%, 59%, and 40% in the AJCC database. The reason for the discrepancy observed in stage II was unclear; however, recently published data in which the same AJCC database was classified using the new eighth Edition classification system^23^ showed similar survival outcomes compared to our results: 5‐year survival for IIA, IIB, and IIC were 98%, 87%, and 82%, respectively. In this new AJCC database analysis, the authors excluded those patients recruited before the SLN biopsy method for classifying stage II disease was introduced, which suggest that the participants with stage II melanoma included in the seventh Edition classification may have included a considerable number of patients with occult LN metastasis.

In this study, we identified that (independent of TNM status) higher age and specific clinical types (NM and MCM) were associated with poor survival as a poor prognostic factor (Table 6). This result confirms previous reports in which these factors were identified as being associated with poor outcome.^3^, ^8^, ^9^ ALM was not associated with survival across the entire cohort; however, regarding stage III, there was significant survival disadvantage for patients with ALM and NM compared with SSM (Figure 1C). Interestingly, survival curves of stage IIIA ALM were significantly worse than that of SSM, whereas no survival difference was found in stage IIIB and IIIC (Figure 3B,C). Because there was no difference regarding T and N subclass frequency in ALM and SSM, ALM itself may have led to worse prognosis in the early stages of lymphatic spread. Further accumulation of stage IIIA patient data is required to confirm this result and to reveal the cause of this difference in prognosis.

As described above, the chances of ALM having BRAF mutation are very low; therefore, immune checkpoint inhibitors should be the most appropriate treatment for advanced ALM. However, the response ratio of Japanese melanoma patients to immune checkpoint inhibitors tended to be lower than that of patients in Western countries.^16^, ^17^, ^18^, ^19^ A South Korean study (a country with a population of a similar ethnical background to Japan) comprised 45.9% ALM and 24.3% MCM diagnoses, reported that only 10.8% of the patients responded to anti‐PD‐1 treatment.^24^ In our previous retrospective study of 45 patients with ALM and MCM treated at our institute, the response rate to anti‐PD‐1 treatment was 12.5% and 25%, respectively.^25^ This study suggested that while the use of anti‐PD‐1 therapy for ALM or MCM did elongate survival, its effect was not durable and most of the patients died of the disease. Moreover, response ratio of ipilimumab for in 60 patients with melanoma who became refractory to nivolumab was only 3.6%,^26^ whereas studies from Western countries report same figure to be 10 to 16%.^27^, ^28^

Such unfavorable outcomes are found not only in the treatment of patients with advanced stages of the disease but are also observed in the adjuvant setting. The CheckMate 238 trial, a randomized phase 3 trial that evaluated the adjuvant use of nivolumab versus ipilimuamb,^29^ reported that patients with cutaneous melanoma (not including ALM and MCM) showed a 39% risk reduction; however, there was no statistical clinical benefit of using nivolumab over ipilimumab for patients with ALM and MCM. Mutation burden has been reported to be associated with response to checkpoint inhibitors^30^; tumors with a higher mutation burden respond to checkpoint inhibitors better than those with a lower mutation burden. ALM and MCM are reported to have a lower mutation burden than other cutaneous melanoma,^31^ which supports the assertion that these clinical types have a lower response ratio than other types. Taken together, this accumulated evidence suggests that ALM and MCM should perhaps be considered as “tough” types of melanoma and therefore require new treatment strategies that are especially designed for these clinical types.

The limitation of this study was the high number of patients whose follow up was lost. In this study, 465 out of 4594 patients (10%) lost follow up and this might cause the bias toward better survival estimates as described in this review.^32^

In conclusion, through our analyses of 4594 patients, we were able to show the current trend of melanoma in Japan, and to the best of our knowledge, this is the largest dataset from an Asian country ever published. It is clear that melanoma occurrence in Japan differs from that of Western countries in a number of respects, for example the high incidence of ALM and MCM found in the Japanese population. We suggest conducting clinical trials to target these rare, but aggressive, types of melanoma.

## CONFLICT OF INTEREST

None declared.
