# Supplementary material for: Clinical and histopathological characteristics and survival analysis of 4594 Japanese patients with melanoma
Source: Cancer Med. 2019 Apr 1;8(5):2146–56. doi: 10.1002/cam4.2110 (PMC6536943; doi:10.1002/cam4.2110)
Supplement: Supplementary file 1 [file CAM4-8-2146-s001.docx]

Supplemental table 1 Collaborating institutes of this study

Department of Dermatology, University of Tsukuba

Department of Dermatology, Shizuoka Cancer Center

Department of Dermatology, Shinshu University School of Medicine

Department of Dermatology, Niigata Cancer Center

Department of Dermatology, University of Nagoya

Department of Dermatology, University of Kyushu

Department of Dermatology, Osaka City University

Department of Dermatology, Saitama Medical University International Medical Center

Department of Dermatology, Kyoto Prefectural University of Medicine

Department of Dermatology, Sapporo Medical University

Department of Dermatology, Kobe University

Department of Department of Dermatology and Plastic Surgery, Kumamoto University

Department of Dermatology, Asahikawa Medical University

Department of Dermatology, Nippon Medical School

Department of Dermatology, Okayama University Graduate School of Medicine

Department of Dermatology, Tohoku University Graduate School of Medicine

Department of Dermatologic Oncology, National Cancer Center Hospital

Department of Dermatology, University of Occupational Environment Health

Department of Plastic Surgery, University of Hokkaido

Department of Dermatology, Saitama Medical University

Department of Dermatology, University of Gifu

Department of Dermatology, Toyama Prefectural Central Hospital

Department of Dermatology, Fukuoka University

Department of Dermatology, Hamamatsu University School of Medicine

Department of Dermatology, Saitama Prefectural Cancer Center

Department of Dermatology, University of Tokyo

Department of Dermatology, Toranomon Hospital

Supplementary table 2 The information collected in the survey

| Category | Items | | | |
| --- | --- | --- | --- | --- |
| Background data | Age | Sex | | Date of admission |
|  | Pregnancy | Skin type | | Other malignancy |
|  | Familial history | Onset to admission | | |
| Primary tumor data | Site | Size | | Clinical type |
|  | Ulceration | Regression | | Clark classification |
|  | Breslow thickness |  | |  |
| Lymph node (LN) | Sentinel LN | LN dissection | | Satellite |
|  | In-transit | Number of nodes with metastasis | | |
| Others | LDH level |  | |  |
| TNM status | T classification | N classification | | M classification |
| Primary therapy | Surgical margin | Adjuvant therapy | | Radiotherapy |
|  | Chemotherapy | Immunotherapy | |  |
| Follow up data | Recurrence | Date of recurrence | | Outcome |
|  | Date of last follow up | | Treatment for recurrence | |
